# Open Abdomen Management and *Candida* Infections: A Very Likely Link

**DOI:** 10.1155/2017/5187620

**Published:** 2017-12-07

**Authors:** Savino Occhionorelli, Monica Zese, Rosario Cultrera, Domenico Lacavalla, Marco Albanese, Giorgio Vasquez

**Affiliations:** ^1^Department of Morphology, Surgery and Experimental Medicine-University of Ferrara and Sant'Anna University Hospital of Ferrara, Ferrara, Italy; ^2^Department of Medical Sciences, Centre for International Cooperation and Development, Infectious Diseases Unit-University of Ferrara and Sant'Anna University Hospital of Ferrara, Ferrara, Italy; ^3^Department of Surgery, Emergency Surgery Service, Sant'Anna University Hospital, Ferrara, Italy

## Abstract

**Objective:**

Laparostomy can be applied in trauma, abdominal sepsis, intra-abdominal hypertension, or compartment syndrome. Systemic infections, especially if complicated by *Candida*, are associated with a high risk of mortality.

**Methods:**

This is a single-centre retrospective case series of 47 cases admitted to our Department, which required laparostomy procedure; we analyzed the type of surgery, temporary abdominal closure, duration of open abdomen, complications, SOFA score, mortality with *Candida* infections, and empirical or targeted antifungal therapy.

**Results:**

We found that patients with *Candida* infection were related with a statistically significant difference (*p* < 0.05) with a complication after OA closure, total complications, time elapsed after OA application, time spent on the first surgical OA application, type of temporary abdominal closure that is used, and duration of the open abdomen. The use of empirical and targeted antifungal therapy is related to the duration of open abdomen too.

**Conclusions:**

Management of the OA is often burdened by sepsis or septic shock, especially when complicated by *Candida* infection. *Candida* score is a validated tool to identify patients who can be treated empirically, but every situation must be considered on an individual basis.

## 1. Introduction

The open abdomen (OA) technique or laparostomy is a surgical option nowadays considered acceptable for the treatment of critically ill patients [1–3]. The key idea is to leave the abdominal cavity open in order to reduce the intra-abdominal pressure in case of abdominal hypertension and/or to allow a better control of the abdominal cavity in case of intra-abdominal infections. Abdominal contents are exposed; thus, they need to be protected with a temporary abdominal closure (TAC) [4]. Several TAC systems are used nowadays [5–8]. Usually, the role of laparostomy is closely linked to damage control surgery, especially in traumatized patients [9]. It can also be adapted in advanced sepsis or in the emergency treatment of acute peritonitis [4, 5, 10–14], in order to prevent or control the frame of septic shock [13, 15]. On this topic, the literature is still being debated nowadays [3, 11]. Fascial closure can be realized <7 days (early) or >7 days (delayed) after the initial OA procedure [5]. The gold standard is the early fascial closure [5, 16–46], in order to reduce complications, but in septic patients, it is less likely to be achieved. Anyway, it should be performed as soon as possible, when abdominal sepsis is under control [5, 17–19]. It is widely reported in the literature that the maintenance of OA predisposes patients to a further microbial and fungal contamination [20]. Therefore, antibiotic and antifungal therapies have an important role for controlling the source of sepsis and the risk of complications during and after laparostomy. Most common complications in OA, like bleeding, anastomosis leakage, EAF appearance, or multiorgan failure (MOF), are linked to sepsis, and they can result in an increased mortality [5, 20, 21]. Several studies have demonstrated the role of invasive *Candida* species (spp.) infection in worsening sepsis or septic shock [22, 23]. Incidence of candidemia varies between 2 and 6.9 per 1000 admissions in ICU irrespective of the diagnosis of admission [24, 25], and it is associated with a high mortality rate, ranging from 35% to 60% [26]. The conditions of the patients can be monitored with outcome prediction models, just like the sequential organ failure assessment (SOFA) [27], in order to correlate the number of dysfunctional organs with the mortality in septic patients [28, 29]. The study evaluates a continuative series of 47 cases treated in the Emergency Surgery Department of Sant'Anna University Hospital of Ferrara with a severe diagnosis of trauma, intestinal ischemia, bleeding, or peritonitis which required OA. It aims at considering the relation between *Candida* infections and diagnosis, duration of the OA, type of TAC used, complications, and outcomes. It also associates this data with microorganism infection and the use of prophylactic or targeted antifungal therapy.

## 2. Methods

In the Emergency Surgery Department of Sant'Anna University Hospital of Ferrara, 44 patients were admitted who required laparostomy (in the opinion of treating surgeon and anaesthesiologist) between February 2010 and September 2016. Among them, 3 patients required a second laparostomy, so a total number of 47 cases are taken into account. A written informed consent was obtained from every patient or from their legal delegates (at the time of intervention or during follow-up). Inclusion criteria were age > 18 years and patients with Björck 2016 Classification System [30] grades 1a, 1b, 2a, 2b, and 2c. Exclusion criteria were age < 18 years, pregnancy, malignancy, patients with Björck 2016 Classification System grades 3a, 3b, and 4. Data were collected by the computerized hospital system and medical records. Source data included preoperative parameters: age, sex, weight, height, admittance diagnosis, and comorbidities. In our Department, different kinds of TAC were used [5]: Bogotà-Bag like (we used a like sterile 3 litre saline bag cut, shaped, and sutured to fascial edges) and Opsite Sandwich technique (we covered the abdomen with a sheet of polyethylene, surrounded by Opsite, abdominal packs, two suction drains, and wall suction). This two techniques could be associated with approaching skin as in skin-only closure technique, realizing a mixed technique and VAC therapy. Opsite Sandwich technique indication, length of the OA (<7/>7 days) [46], and number and distance of relooks were also considered. The study analyzed data in relation to ileus, colon, or intra-abdominal parenchymal organs (liver, pancreas, or kidney) involved. In the considered cases, the stomach was never affected. Complications have been analyzed following the Clavien-Dindo classification [31]. In our study, we have considered bleeding which required blood transfusion (grade IIa), intestinal ischemia (grade IVb), anastomosis leakage (grade IIIb), post operatory bilious fistula (grade IIIa), entero-atmospheric fistula (EAF) (grade IIIa), wound dehiscence (grade IIa), and MOF (grade IVb) among OA relooks and after OA definitive closure. These parameters were correlated with bacterial and *Candida* infections. Microorganisms were analyzed instead of detected with bronchial, urine, blood, and peritoneal analyses in every patient. Data were also related to time spent after OA application and appearance of *Candida* infections (<7/>7 days) and antimicrobial and antifungal therapies in both ICU and Surgical Department with a follow-up of 2 months. Organ dysfunction was evaluated using the SOFA score. Every case was classified following 3 degrees of SOFA score steps in relation to mortality risk: <3, 3–9, and >9. For trauma, we used the Injury Severity Score (ISS) classification [32]. Data were analyzed with statistic chi-square test, considering *p* < 0.05 as statistically significant.

## 3. Results

**Figure 1:**
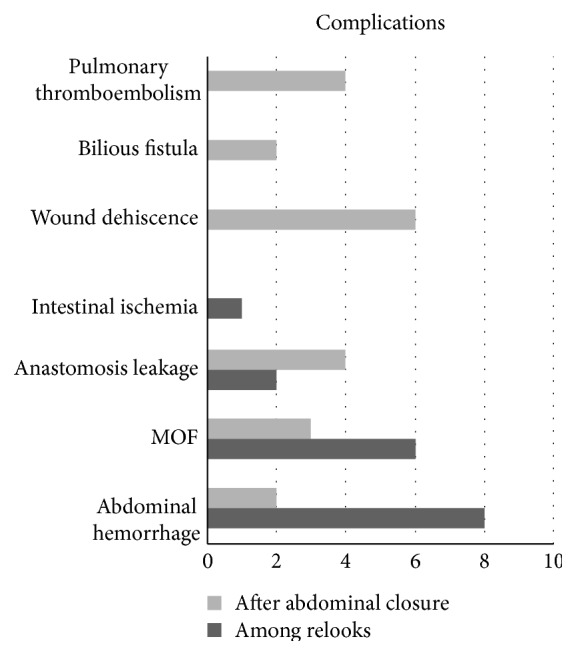
Complications among relooks and after abdominal closure: in the picture are evidenced principal complications among and after abdominal relooks.

**Figure 2:**
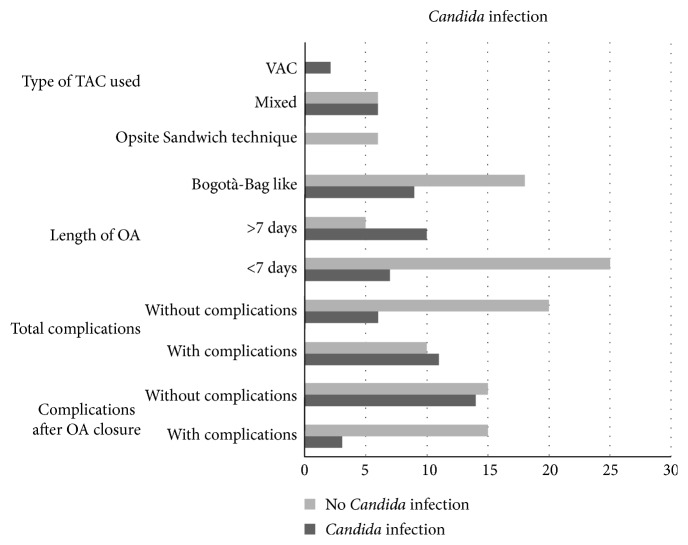
Correlation among *Candida* infection and type of TAC used, duration of OA, total complications, and complications after OA closure.

The study included 47 cases of OA performed in 44 patients. Three patients, all males, required a second laparostomy after the closure of the previous one: two for peritonitis and one for abdominal bleeding. Of 44 patients, 15 were females (34%) and 29 were males (66%) with a female : male ratio of 1 : 1.9. The average age was 63 years (median 68, range 24–86), and the average ages of women and men were 69.7 and 58.8, respectively. Average BMI was 28.7 kg/m^2^ (range 17–47 kg/m^2^). Comorbidities and ASA score [33] are shown in Table 1. We admitted 27/44 patients from triage; 17/44 patients were already hospitalized in other Hospital Departments (Table 2). All cases have been analyzed considering ileal, colonic, or other intra-abdominal parenchymal organ involvement (Table 2). The 6 traumatized patients were evaluated in triage using the ISS score: the average value was 28.8 (range 21–38, median 27). The study of statistical correlation showed the presence of a significant relation between the concerned intestinal tract and the application of OA (*p* < 0.05). OA was applied to prevent IAH in 21/47 cases (44.7%), while it was performed in the presence of IAH in the other cases 26/47 (55.3%) [34]. The SOFA score was <3 in 8/47 cases (17%), 3–9 in 15/47 (31.9%), and >3 in the other 24/47 cases (51.1%), so in 83% of cases, there was a high risk of mortality related to sepsis. We also evaluated the duration of the OA maintaining (<7 or >7 days) [5, 17] and the type of OA used [35]. In 32/47 cases (68.1%), OA was kept <7 days, while only in 15/47 (31.9%), OA was kept over 7 days. We used Bogotà-Bag like in 27/47 cases (57.4%), Opsite Sandwich technique in 6/47 cases (12.8%), and mixed technique (Bogotà-Bag like or Opsite Sandwich technique + skin-only closure) in 12/47 cases (25.5%). VAC therapy was used only twice (4.3%). Considering the 3 patients treated after a previous laparostomy, 2 of them kept OA < 7 days and only one > 7 days. In all 3 cases and in both laparostomies, we exploited Bogotà-Bag like TAC. Anastomosis was done in 34/47 cases (77.3%), 13/47 at the first look (27.66%) and 21/47 at the following relooks (44.68%). In 13/47 cases (27.66%), a temporary or definitive stoma was created. Only 3/47 cases (6.4%) developed EAF [36]. Almost always, but 4 cases (7.2%), abdominal wall was closed by direct closure. In 4 cases, the closure was done by a biological prosthesis; of them, 2 cases (1.8%) also needed a component separation technique [37]. Complications appeared in 29/47 cases (61.7%). Complications among relooks were 17/47 (36%): abdominal hemorrhage in 8 cases (17%), development of MOF in 6 cases (12.7%), anastomosis leakage in 2 cases (4.2%), and caecum ischemia in 1 case (2.1%). Complications after OA closure appeared in 21/47 cases (44.7%): hemorrhage in 2 cases (4.3%), anastomotic leakage in 4 cases (8.5%), wound dehiscence in 6 cases (12.8%), MOF in 3 cases (6.4%), and bilious fistula in 2 cases (4.3%). Furthermore, in 4 cases, pulmonary thromboembolism was detected and treated adequately (in Figure 1, complications are shown among relooks and after abdominal closure). Several types of bacteria were found in blood analysis in 19/47 cases (40.4%) and in peritoneal analysis in 32/47 cases (68.1%). Sometimes, different kinds of microorganisms were found in different cultural analyses of the same case. The bacterial population was very heterogeneous and therefore not sufficient to allow for a statistically significant analysis. Principal bacteria species and their findings are shown in Table 3. *Candida* spp. were detected in 17/47 cases (36.2%) and in several cases, also in different analyses of the same patient (Table 4). *Candida* infection (revealed by blood and/or peritoneal cultures) appeared in 15/17 cases (88.2%). In the remaining 2/17 cases (11.8%), *C. parapsilosis* and *C. albicans* were evidenced in bronchial analysis only and they had not been treated with an antifungal therapy. Bronchial Candida contamination was not treated in agreement with the infectious specialist because of a single colonization. Empirical antifungal therapy was established at the moment of admission in ICU in 7/47 cases (14.9%), and only 4/47 (8.6%) of them developed a subsequent *Candida* infection. In 3 of these cases, *C. albicans* was detected in peritoneal fluid analysis, and in the latter case, *C. glabrata* and *C. albicans* were found in peritoneal analysis and at the same time *C.* parapsilosys in urine analysis. In 11/47 cases (23.4%), a targeted therapy was applied only when cultures detected *Candida* spp. In our study, there was no evidence of correlation between *Candida* infection and the three different grades of SOFA score, near-operative death, the indication of OA application, complications among relooks, different intestinal involvements, comorbidities (such as obesity, renal pathologies, diabetes, and immune suppression), and the relation between IAH and the creation of anastomosis or stoma (*p* > 0.05) (Table 5). The overall mortality in our study is 13/44 patients (29.5%): 4 women (9.1%) and 9 men (20.5%), 6 of them (13.6%) with invasive *Candida* infection. Overall, 6 of the 17 patients affected by *Candida* infections died (35.3%). In one case, there was only bronchial *Candida* contamination; in 3 cases, targeted therapy was applied; and in 2 cases, patients were treated with empirical antifungal therapy. We found a relation between *Candida* infection and both complications after OA closure and total complications. We also considered the elapsed time after the OA application and the first positive analysis for *Candida* spp. (<7 days or >7 days). We found a statistically significant relation between time spent on the first surgical OA application and *Candida* infection appearance. There was also a statistically significant relation between *Candida* infection and the duration of the OA. We found a correlation between *Candida* and the type of TAC used. This analysis also considered the two cases of VAC therapy OA, even though they are not to be considered statistically significant. More infections occurred with the use of Bogotà-Bag like; no infections occurred with Opsite Sandwich technique and a provided equal in mixed technique (Table 6, Figure 2). However, we did find a statistically significant relation between antifungal therapy and the total duration of the OA. In particular, antifungal therapy was not used in the majority of OA which were kept <7 days. Data showed that *Candida* infection is less frequent when the duration of the OA is <7 days, as well as the setting of empirical antifungal therapy. Conversely, when OA is kept >7 days, antifungal therapy is used in more cases (Table 6, Figure 2).

## 4. Discussion

Many aspects must be considered when OA is used, such as the reason for application of the laparostomy, age, and comorbidities of the patient, septic status, and hydration conditions [5]. As revealed by Cristaudo et al. [36], currently, there are no published reviews of prognostic factors with regard to definitive fascial closure, mortality, and intra-abdominal complications of patients being managed with OA. Intra-abdominal infections are considered as emergencies, and a successful outcome depends on early diagnosis, an appropriate surgical treatment, and adequate antibiotic therapy [47]. The control of invasive *Candida* infections is related to a reduced mortality [23, 38, 47]; nevertheless, criteria for starting an empirical antifungal therapy are poor. In many cases, unnecessary starting of antifungal therapy can cause resistance [39, 40]. The colonization rate of *Candida* spp. reaches up to 80% in patients who reside in intensive care units (ICUs) more than a week, and the mean rate of development of invasive disease is 10% in colonized patients [48]. *Candida* colonization is considered multifocal when the same or two different species are found in two or more nonadjacent loci of the organism, such as urine or bronchial expectorate [23]. As reported by Leroy et al. in [23], invasive *Candida* infection was defined by at least one positive blood culture or peritonitis, diagnosed by macroscopic findings and direct examination or positive culture for *Candida* in the peritoneal fluid collected during a surgical procedure. Invasive candidemia can be defined using the criteria proposed by Leon et al. in the “*Candida* score” [41]. Components of the *Candida* score are severe sepsis, total parenteral nutrition, surgery, multifocal *Candida* colonization, invasive mechanical ventilation, central venous catheter, urinary catheter, antibiotic therapy > 5 days in the last 2 weeks, renal replacement therapy, insulin-dependent diabetes, and immune suppression [23]. In our study, the SOFA score was >3 in 39/47 cases (83%) and total parenteral nutrition was always set at admission in ICU, just like invasive mechanical ventilation, central venous catheter, and urinary catheter. In cases where we found multifocal colonization, we considered diabetes, immune suppression, renal diseases, and previous antimicrobial therapy without finding a statistically significant relation with *Candida* infection. *Candida* infection was not significantly related with immune suppression and insulin-dependent diabetes. As reported by Leroy et al. [23], criteria for starting empirical antifungal therapy in ICU patients are poorly defined and recent IDSA guidelines suggesting that “empirical antifungal therapy should be considered in critically ill patients with risk factors for invasive candidiasis and no other known cause of fever” could lead to an overuse of antifungal agents. Despite the literature is still being debated on this topic, the “*Candida* score” can be considered an effective tool to guide clinicians in applying antifungal therapy. In our case series, empirical therapy was set in 7 cases. In 2 cases, *Candida* was found in bronchial expectorate without fever or multiple colonization, so it was not treated, in agreement with the infectious specialist. Targeted therapy was applied in 11 cases after the detection of *Candida* spp. in peritoneum or in blood analysis. The definitive decision to empirically treat *Candida* is left at the single anaesthesiologist in our Department. Some studies, as [20, 42], depict how microbial colonization evolves during OA maintenance. *Candida* infection is not an exception, and the risk of the development increases with the patient's vulnerability [43]. In our case series, we underlined that a longer OA maintenance increases the risk of developing *Candida* infection. In [47], it is reported that *Candida* peritonitis is burdened by a high mortality rate (about 38%) and our case series attests to these values. Anyway, in our study, we must consider some biases. First of all, the small number of patients was considered, who developed *Candida* infection. Furthermore, there were 2 patients which required VAC therapy. However, in both cases, EAF appeared; therefore, we decided to not use this device in further cases. Secondly, our study is encumbered by the fact that during the admission, in conditions of urgency, our Department works with surgeons of different teams; hence, the decision to create OA and type of TAC is left at the discretion of the relevant surgeon. The great heterogeneity of bacteria detected in blood and/or peritoneal analysis could be indicative for sample contamination or colonization as confirmed by the poor efficacy of antibiotic therapy to resolve the post operative infective complications. Moreover, we showed the good response to antifungal therapy. On the other hand, an important bias has originated due to the possibility that the patient's vulnerability could be linked to previous or actual antibiotic therapies or concomitant infections. So we decided to evaluate only *Candida* infection and OA management, with the aim of understanding the presence or absence of a statistically significant relation. We are aware that our data need to be integrated into a more complex analysis system. For this reason, our Department has taken part in the IROA multicenter study [44, 45] and plans to realize a prospective study considering OA management and *Candida* infection appearance. The results of our case series showed that the use of different TACs, even Bogotà-Bag like, cannot be discouraged, confirming the results of the recent IROA multicenter study [44]. The choice of exploiting OA not only in traumatized patients, but also in peritonitis and sepsis, is a working treatment in which we believe. Our idea is also enforced by an unchanged death rate in a framework that is burdened by a high mortality rate itself [44] in a high septic risk (SOFA score in fact was >3 in 83% of cases). Surely, this is a case series which concerns a small number of patients in a single centre, but it can represent a starting point for a better and more standardized way of treatment in different situations regarding serious diagnosis. Statistically significant relations may be altered by the reduced number of cases, and our study can be considered as an initial assessment of the relation between *Candida* infection and the various aspects of laparostomy. However, it remains an initial study. Our follow-up deliberately analyzes short distance of time, to understand which complications can occur during near-operative time.

## 5. Conclusions

OA technique and its use in critical situations are still hotly debated in the literature. No matter how seemingly effective, the annexed life-treating complications, which require highly experience in surgical, infectious, and anesthesiology treatments, must be considered when using OA. The right choice of TAC and the early fascial closure of the abdomen appear to be essential to avoid additional risk situations. *Candida* infection in debilitated patients, as well as patients who are subjected to OA, implies a great risk to mortality. For this reason, the treatment of invasive *Candida* infection should be based on predictive models, such as the “*Candida* score” without, in our opinion, forgetting to consider the needs and the characteristics of every individual patient, in order to avoid over- or underestimated treatment. Of course, much remains to be done to achieve optimal results in the treatment of this critical situation.

## Figures and Tables

**Table 1:** Comorbidities and ASA score.

Comorbidities	*N*	%
Hypertension	21	44.7
Heart diseases	16	34.0
Renal diseases	9	19
Pneumological disorders	3	6.4
Previous cancer	7	14.9
Immune suppression	1	2.1
Diabetes	7	14.9
Obesity	23	48.9
ASA score		
I	2	4.3
II	7	14.9
III	19	40.4
IV	17	36.2
V	2	4.3

**Table 2:** Cases admitted, diagnosis, and abdominal involvement.

Diagnosis	Trauma/intestinal bleeding	Ischaemia	Peritonitis
Triage	6 (12.76%)	9 (19.14%)	12 (25.6%)
Already hospitalized	5 (10.6%)	7 (14.9%)	8 (17%)
Total	11 (23.4%)	16 (34%)	20 (42.6%)
*Intestinal involvement*			
Ileus	4 (8.5%)	11 (23.4%)	3 (6.4%)
Colon	3 (6.4%)	5 (10.6%)	10 (21.3%)
Liver, spleen, kidney	3 (6.4%)	0 (0%)	5 (10.6%)
Reinterventions	1 (2.1%)	0 (0%)	2 (4.2%)

**Table 3:** Different bacteria in blood and peritoneal analysis. We considered the total where we found the presence of bacteria, and we divided them in base of blood or peritoneum detection.

Bacteria	*E. coli*	*Klebsiella pneumoniae*	*Enteorococcus faecalis*/*faecium*	*Pseudomonas aeruginosa*	Staphylococcus/streptococcus
Total cases	22 (46.8%)	10 (21.3%)	21 (44.7%)	7 (14.9%)	29 (61.7%)
Peritoneum	19 (40.4%)	9 (19.1%)	16 (34%)	5 (10.6%)	21 (44.7%)
Blood	10 (21.3%)	2 (4.3%)	12 (25.5%)	5 (10.6%)	16 (34%)

**Table 4:** Different *Candida* species and their findings.

*Candida*	*parapsilosis*	*albicans*	*glabrata*	*mirabilis*	*tropicalis*
Bronchial	1 (5.9%)	4 (23.5%)	1 (5.9%)	0	0
Urine	1 (5.9%)	3 (17.6%)	1 (5.9%)	1 (5.9%)	1 (5.9%)
Peritoneum	0	10 (58.8%)	3 (17.6%)	0	0
Blood	0	2 (11.8%)	0	0	0

**Table 5:** Not statistically significant (*p* > 0.05) and % among *Candida* infection, the three different grades of SOFA score, near-operative death, the indication of OA application, complications among relooks, different intestinal involvements, and the relation between IAH and the creation of anastomosis or stoma.

		*Candida* infection				
		Yes	No	% at total cases (47)		*p* > 0.05
Sofa score	<3	6	2	2.82	0.94	0.42
	3–9	7	8	3.29	3.76	
	>9	14	10	6.58	4.7	

Mortality rate	Dead	7	6	3.29	2.82	0.59
	Alive	20	24	9.4	11.28	

OA indication	Trauma bleeding	6	5	2.82	2.35	0.95
	Ischemia	9	7	4.23	3.29	
	Peritonits	12	8	5.64	3.76	

Complications among relooks	With complications	10	7	4.7	3.29	0.88
	Without complications	17	13	7.99	6.11	

Intestinal involvement	Ileus	12	7	5.64	3.29	0.77
	Colon	10	8	4.7	3.76	
	Liver, spleen, kidney	5	5	2.35	2.35	

IAH	Yes	14	12	6.58	5.64	0.57
	No	13	8	6.11	3.76	

Anastomosis	Yes	19	15	8.93	7.05	0.72
	No	8	5	3.76	2.35	

Stoma	Yes	8	3	3.76	1.41	0.24
	No	19	7	8.93	3.29	

**(a):** 

		*Candida*				
		Yes	No	Total cases (47) at *p* < 0.05 and %		*p* < 0.5
Total complications	Case positive	3	15	6.40	32	0.03
	Case negative	14	15	29.60	32	

Complications after OA closure	Case positive	11	10	23.40	21.28	0.03
	Case negative	6	20	12.77	42.55	

Duration of OA	<7 days	7	25	14.89	53.19	0.003
	>7 days	10	5	21.28	10.64	

Type of TAC used	Bogotà-Bag like	9	18	19.15	38.30	0.04
	Opsite Sandwich technique	0	6	0.00	12.77	
	Mixed	6	6	12.77	12.77	
	VAC	2	0	4.26	0.00	

**(b):** 

		<7 days	>7 days	Total cases (47) at *p* < 0.05 and %		*p* < 0.5
Duration of OA	<7 days	3	3	6.38	6.38	0.01
	>7 days	4	7	8.51	14.89	
	No infection	25	5	53.19	10.64	

**(c):** 

		OA length of stay				
		<7 days	>7 days	Total cases (47) at *p* < 0.05 and %		*p* < 0.05
Antifungal	Not used	22	4	46.81	8.51	0.02
	Empirical	4	6	8.51	12.77	
	Targeted	6	5	12.77	10.64	

## Abbreviations

OA: Open abdomen
IAH: Intra-abdominal hypertension
ACS: Abdominal compartment syndrome
TAC: Temporary abdominal closure
SOFA: Sequential organ failure assessment score
EAF: Entero atmospheric fistula
MOF: Multiorgan failure
*C.:*: *Candida*
spp.: Species
ISS: Injury Severity Score
ASA: American Society of Anesthesiologist Score.

## Additional Points

*Availability of Data and Materials*. Data were collected by the computerized hospital system (SAP Front End© and Ormaweb©) and medical records. The datasets used and analyzed during the current study are available from the corresponding author on a reasonable request. All data generated or analyzed during this study are included in this published article.
